# Genome-Wide Analysis of *Staphylococcus aureus* Sequence Type 72 Isolates Provides Insights Into Resistance Against Antimicrobial Agents and Virulence Potential

**DOI:** 10.3389/fmicb.2020.613800

**Published:** 2021-01-20

**Authors:** Nayab Batool, Amen Shamim, Akhilesh Kumar Chaurasia, Kyeong Kyu Kim

**Affiliations:** ^1^Department of Precision Medicine, Sungkyunkwan University School of Medicine, Suwon, South Korea; ^2^Institute of Antimicrobial Resistance and Therapeutics (IAMRT), Sungkyunkwan University (SKKU), Suwon, South Korea; ^3^Samsung Advanced Institute for Health Sciences and Technology (SAIHST), Samsung Medical Center (SMC), Sungkyunkwan University School of Medicine, Seoul, South Korea

**Keywords:** Staphylococcus aureus, sequence type 72, antibiotics resistance, virulence factors, subtractive genomics

## Abstract

*Staphylococcus aureus* sequence type 72 (ST72) is a major community-associated (CA) methicillin-resistant *Staphylococcus aureus* (MRSA) that has rapidly entered the hospital setting in Korea, causing mild superficial skin wounds to severe bloodstream infections. In this study, we sequenced and analyzed the genomes of one methicillin-resistant human isolate and one methicillin-sensitive human isolate of ST72 from Korea, K07-204 and K07-561, respectively. We used a subtractive genomics approach to compare these two isolates to other 27 ST72 isolates to investigate antimicrobial resistance (AMR) and virulence potential. Furthermore, we validated genotypic differences by phenotypic characteristics analysis. Comparative and subtractive genomics analysis revealed that K07-204 contains methicillin (*mecA*), ampicillin (*blaZ*), erythromycin (*ermC*), aminoglycoside (*aadD*), and tetracycline (*tet38*, tetracycline efflux pump) resistance genes while K07-561 has ampicillin (*blaZ*) and tetracycline (*tet38*) resistance genes. In addition to antibiotics, K07-204 was reported to show resistance to lysostaphin treatment. K07-204 also has additional virulence genes (*adsA*, *aur*, *hysA*, *icaABCDR*, *lip*, *lukD, sdrC*, and *sdrE*) compared to K07-561, which may explain the differential virulence potential of these human isolates of ST72. Unexpectedly, the virulence potential of K07-561 was higher in an *in vivo* wax-worm infection model than that of K07-204, putatively due to the presence of a 20-fold higher staphyloxanthin concentration than K07-204. Comprehensive genomic analysis of these two human isolates, with 27 ST72 isolates, and *S. aureus* USA300 (ST8) suggested that acquisition of both virulence and antibiotics resistance genes by ST72 isolates might have facilitated their adaptation from a community to a hospital setting where the selective pressure imposed by antibiotics selects for more resistant and virulent isolates. Taken together, the results of the current study provide insight into the genotypic and phenotypic features of various ST72 clones across the globe, delivering more options for developing therapeutics and rapid molecular diagnostic tools to detect resistant bacteria.

## Introduction

*Staphylococcus aureus* is a commensal organism of the skin and inhabitant of the nares in nearly 30% of the global human population (Krismer et al., 2017). *S. aureus* has the potential to cause both mild skin infections and fatal diseases (soft-tissue infections, pneumonia, bacteremia, sepsis, and endocarditis) with mortality higher in those subjects with a compromised immune system (Fournier and Philpott, 2005; McDanel et al., 2016). Community-associated methicillin-resistant or -sensitive *S. aureus* (CA-MRSA/MSSA) sequence type 72 (ST72) is a prevalent isolate in South Korea (Joo et al., 2012). The long-standing perception that ST72 isolates have low levels of virulence and resistance has recently been challenged by the finding that these isolates are resistant to desiccation and can adapt to hypotonic solutions, both of which can promote their survival in a hospital setting (Joo et al., 2017). CA-MRSA ST72 has evolved resistance to multiple antimicrobial agents through various mechanism (Stapleton and Taylor, 2002; Sutcliffe and Leclercq, 2002; Blair et al., 2015; Munita and Arias, 2016; Varona-Barquin et al., 2017; Moreno-Flores et al., 2018). Methicillin, vancomycin-intermediate, and vancomycin-resistant *S. aureus* (MRSA, VISA, and VRSA, respectively) ST72 clones are life-threatening antimicrobial-resistant strains that are extremely challenging to treat clinically (Chung et al., 2012). CA-MRSA ST72 has rapidly entered the hospital setting as hospital-associated (HA) multiple drug resistant (MDR) isolates causing blood-stream infections (Tsukimori et al., 2014). It is important to further our understanding of the genotypic and phenotypic features of various ST72 clones across the globe to assess current and future therapeutic options, to facilitate rapid molecular diagnosis, and to allow effective measures to be devised to reduce their rapid dissemination. However, no genome-wide comprehensive analyses of the genotypic and phenotypic correlations between antibiotic resistance and acquisition of isolate-specific virulence genes has been performed. In addition, the variability in infection potential and dissemination of ST72 clones across the globe have not been characterized.

Sequence type 72 isolates have previously been typed using pulse-field gel electrophoresis (PFGE; Melles et al., 2007; Potel et al., 2016), while the antimicrobial resistance of ST72 isolates has previously been studied by treating a few local isolates of MRSA/MSSA ST72 from Korea with various antibiotics (Ko et al., 2011). The rapid dissemination of CA-MRSA ST72 isolates into the hospital setting spurred scientists and clinicians to sequence the ST72 genome of *Staphylococcus aureus* CN1 (CP003979.1; Chen et al., 2013) to investigate the adaptability of ST72 isolates in the context of HA ST72-MRSA infections. The emergence of a vancomycin resistant ST72 isolate has led to a focus on alternative therapeutics to treat HA-MRSA infections, especially in the intensive care unit (ICU; Varona-Barquin et al., 2017). Lysostaphin, a metalloendopeptidase secreted from *Staphylococcus simulans* biovar *staphylolyticus*, cleaves the pentaglycine bridge of peptidoglycans in the cell wall (Browder et al., 1965) and has previously been shown to possess high killing-kinetics against various sequence types of MSSA/MRSA (Huber and Huber, 1989; Grunenwald et al., 2018; Kim et al., 2019), and thus is considered an efficient alternative for treatment of MDR CA/HA *S. aureus* infections. One of the human isolates, K07-204, showed much higher lysostaphin resistance (*lys^r^*) than the human isolate K07-561, which we characterized as lysostaphin susceptible (*lys^s^*; Batool et al., 2020a,b). Therefore, we analyzed the genotypic and phenotypic characteristics of these two (K07-204 and K07-561) isolates to assess possible mechanisms of resistance to antimicrobial agents and identified isolate-specific virulence factors in comparison to other ST72 isolates and a community-associated *S. aureus* USA300 (ST8; Diep et al., 2006).

In this study, the genomes of 27 additional isolates of ST72 along with K07-204 and K07-561 were selected based on varying time of isolation and locations across the globe (Supplementary Table S1). The comparative and subtractive genomics was applied to evaluate the variability in antimicrobial resistance and virulence potential of K07-204 and K07-561 isolates of ST72. The genome-wide analyses provided the isolate-specific antibiotics resistance and virulence genes among various ST72 isolates which could be useful in determining the current and future therapeutic options and facilitate rapid molecular diagnosis of ST72 isolates.

## Materials and Methods

### Bacterial Cell Growth Conditions

*Staphylococcus aureus* ST72 isolates and *Staphylococcus aureus* USA300 FPR3757 (hereafter denoted as SAUSA300) were grown on tryptic soy agar (TSA) plates at 37°C overnight. Bacterial broth cultures were grown by inoculating tryptic soy broth (TSB) media with a single colony from a TSA plate. The bacterial broth culture was grown under orbital shaking (200 rpm) culture conditions at 37°C for 14–16 h. Bacterial cell growth was monitored by measuring optical density at 600 nm (OD_600_) using a spectrophotometer (GE Healthcare, United States). Bacterial cells were harvested by centrifugation (3,220 × *g*) at 4°C followed by washing with 1X phosphate buffered saline (PBS, pH 7.2).

### Genome Analysis of *S. aureus* Isolates

**Figure 1:**
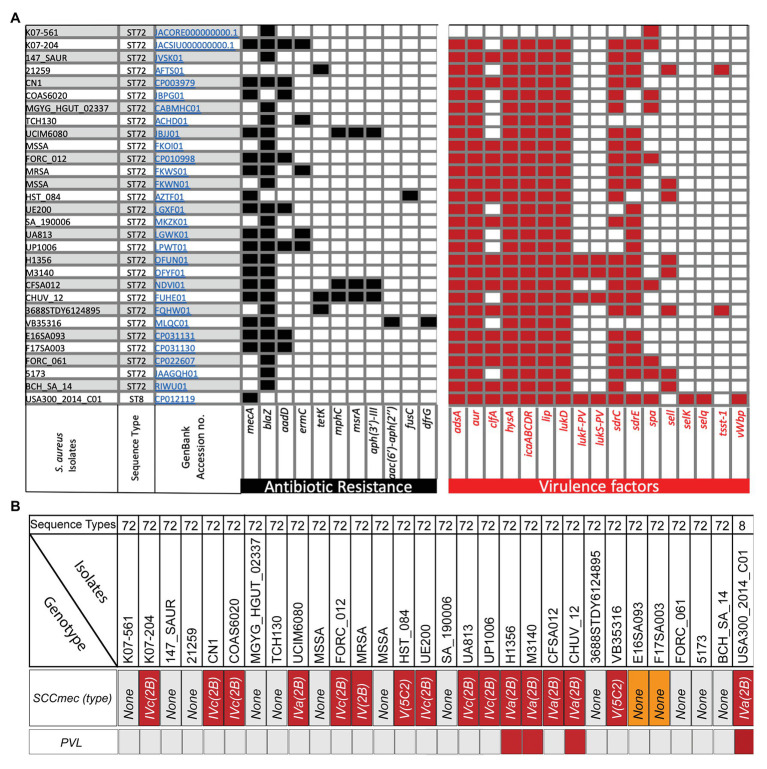
Analysis of isolate-specific distribution of antibiotic resistance and important offensive-virulence genes along with *SCCmec* and Panton Valentine leucocidin (PVL) in various sequence type 72 (ST72) isolates obtained at different times and locations. **(A)** Analysis of antibiotics genes in ST72 K07-204 and K07-561 along with 27 other ST72 isolates and ST8 (SAUSA300) revealed variability in their antibiotic resistance. Subtractive genomics allowed identification of isolate-specific virulence genes. The GenBank Accession numbers of each isolates are hyperlinked and ST is indicated in the corresponding rows; **(B)** Analysis of *SCCmec* cassette and PVL in various isolates of ST72 and SAUSA300 (ST8). The analysis showed that out of 29 ST72 isolates, only three ST72 isolates are PVL positive while 15 ST72 isolates showed the presence of *SCCmec* cassette ranging from type *IV(2B)*, *IVa(2B)*, *IVc(2B)* to *V(5C2)*. ST72 isolates E16SA093 and F17SA003 showed the presence of *mecA* gene, but no typable *SCCmec* cassette was identified (orange box).

Draft genomes of human isolates of ST72, K07-204 and K07-561 (Korea) are publicly available under GenBank accession numbers JACSIU000000000.1 and JACORE000000000.1, respectively. The genomes of the K07-204 and K07-561 were announced wherein the methods related to whole genome sequencing, genome assembly and annotation were described (Batool et al., 2020a). Genome sequences of other *S. aureus* STs used in this study were downloaded from GenBank. The GenBank accession numbers of the genomes of the 27 additional ST72 isolates and *S. aureus* USA300 are listed in Figure 1 and Supplementary Table S1 with their time and country of isolation/genomic data availability.

### Comparative and Subtractive Genomics

Genes responsible for antibiotic resistance and virulence were identified by analyzing the whole genomes of K07-204 and K07-561 along with the genomes of 27 other ST72 isolates and *S. aureus* USA300 (ST8). Genome-wide total virulence factors present in each isolate were analyzed using BacWGSTdb (Ruan and Feng, 2016; Ruan et al., 2020). Specific virulence factors were identified by a subtractive approach wherein total virulence factors were subtracted from common virulence factors present in every isolate/sequence type to identify isolate-specific virulence factors (Supplementary Table S1).

### Assessment of Antimicrobial Resistance

To validate isolate-specific antimicrobial resistance obtained by genomic analyses of K07-204 and K07-561, the resistance/susceptibility phenotypes against methicillin (MIC_90_ 64 μg/ml; Lee and Chong, 1996), ampicillin, erythromycin, kanamycin, and tetracycline were assessed in Mueller-Hinton broth (MH broth) using Clinical and Laboratory Standard Institute (CLSI) protocols. The resistance/susceptibility of the ST72 isolates were defined based on the CLSI document M100 and European Committee on Antimicrobial Susceptibility Testing (EUCAST) clinical breakpoint table v.10.0. The differential response to lysostaphin of K07-204 and K07-561 was tested as described earlier (Kim et al., 2019; Batool et al., 2020a). Varying concentrations of lysostaphin (1, 1.5, and 2 U) were used to evaluate the cut-off value of lysostaphin to determine the lysostaphin resistant/susceptible phenotype of ST72 isolates (Batool et al., 2020a). Briefly, the overnight grown cells of K07-204 and K07-561 were washed once with 1X PBS (pH 7.2), and turbidity of the culture (OD_600_) was maintained to one. Cells were treated with 2 units of lysostaphin for 5 min, and various dilutions were plated to enumerate colony forming unit (CFU).

### Staphyloxanthin Assessment

Staphyloxanthin pigment was extracted using a previously published protocol (Abbas et al., 2019). Briefly, *S. aureus* strains were grown in TSB for 12 h at 37°C and 200 rpm orbital shaking culture conditions. Cells were collected by centrifugation and resuspended in 0.2 ml methanol. After complete mixing *via* vortexing, cells were warmed at 55°C for 3 min. Methanolic extracts of staphyloxanthin were separated by centrifugation to obtain a clear methanolic extract devoid of cell debris. Absorbance of the extracted staphyloxanthin was measured at 465 nm (A_465nm_; Tecan, Infinite M200, Switzerland).

### *In vitro* Infection Experiments

The infection potential of the ST72 isolates was compared with that of wild type SAUSA300 using *in vitro* infection experiments. Specifically, invasion potential and phagocytosis followed by intracellular cell survival were assessed using HEK293 and RAW264.7 cell lines, respectively. Mammalian cells were grown in Dulbecco’s modified eagle medium (DMEM) supplemented with 10% fetal bovine serum (FBS) in a 5% carbon dioxide humidified incubator at 37°C. Cells were transferred to six-well plates (1 × 10^6^ cells/well) and incubated for 24 h. After 24 h of growth, the spent media was removed, and cells were provided with infection medium (DMEM without FBS) 2 h before infection. HEK293 and RAW264.7 cells were infected with SAUSA300 at a multiplicity of infection of 10 (*moi*, 10) for 30 min as described previously (Kim et al., 2019). Extracellular cells were killed using lysostaphin (5 U, 5 min) or gentamicin (400 μg/ml, 60 min). Mammalian cells infected with *S. aureus* were washed three times with 1X PBS to remove residual antibacterial agent. Cells were trypsinized and collected by centrifugation. Cell pellet was treated with 0.04% Triton-X100 to lyse mammalian cells to recover intracellular bacterial cells. Intracellular bacterial cells were diluted in 1X PBS and bacterial count was assessed by plating 100 μl of various dilutions of the bacterial suspension on TSA plates to enumerate CFU.

### *Galleria mellonella* Infection Model

Wild-type SAUSA300 and ST72 (K07-204 and K07-561) isolates were grown overnight in TSB media under orbital shaking (200 rpm) culture conditions at 37°C. A 100-fold dilution of the overnight cultures was reinoculated into TSB media for 6 h. Bacterial cells were collected by centrifugation (3,220 × *g*, 4°C) and washed once with 1X PBS. Cell number was maintained by adjusting the optical density (OD_600_) to 1 in 1X PBS. A *Galleria mellonella* (wax-worm) infection model was used as described earlier (Imdad et al., 2018), with slight modifications for *S. aureus*. Briefly, *G. mellonella* worms were utilized within a week of their receipt. Worms were injected with 2 × 10^5^ cells (20 μl) in the last left posterior leg using a 0.3 ml syringe (Becton Dickinson and Company, United States) and kept at 37°C for observation. In each experiment, a group of worms were injected with 20 μl of 1X PBS as the placebo control group. Survival of wax-worms was assessed at different time intervals and the experiment was terminated within 60 h of injection.

### Phylogenetic and Dissemination Analyses

The genomes of various STs were retrieved from NCBI GenBank. Single nucleotide polymorphisms (SNPs) were assessed; and phylogenetic trees were constructed using the software packages CSI phylogeny and iTOL (Letunic and Bork, 2007; Kaas et al., 2014). SNP tables for the various ST72 strains used in the study were analyzed using the CSI phylogeny server.^1^ Dissemination of various ST72 isolates was analyzed based on the year of isolation and locations reported in the GenBank submission reports (BIOSAMPLES/BIOPROJECTS) and scientific literature.

## Results and Discussion

### Genotypic and Phenotypic Studies of Antimicrobial Resistance in Human Isolates of ST72

#### Distributions of Various Antibiotics Resistance Genes in ST72 Isolates

For the analysis of antibiotics resistance genes in ST72 isolates, we have chosen 27 ST72 isolates (Supplementary Table S1) based on the year of isolation and locations in addition to K07-204 and K07-561. In addition, *S. aureus* USA300 has also been analyzed since it is highly virulent and is known to possess variable levels of resistance toward antibiotics including methicillin (Thurlow et al., 2012). Genome-wide analysis of the presence of antibiotic resistance genes in 29 ST72 isolates including K07-204 and K07-561 in comparison with *S. aureus* USA300 (GenBank Accession no. CP012119) revealed the presence and distribution of 10 additional antibiotics genes, namely *blaZ*, *aadD*, *ermC*, *tetK*, *mphC*, *msr(A)*, *aph(3')-III*, *aac(6')-aph(2')*, *fusC*, and *dfrG* genes conferring the resistance against their corresponding antibiotics (Figure 1A). The *blaZ* gene encoding β-lactamase was present in the majority (~86%) of ST72 isolates, indicating widespread resistance of ST72 isolates to penicillin (Figure 1A). The *mecA* structural gene conferring methicillin-resistance (Stapleton and Taylor, 2002) was found to be present in both *S. aureus* USA300 (ST8; Diep et al., 2008) and ST72 isolates, with the gene present (MRSA) in some isolates and absent (MSSA) in others (Figure 1). K07-561 did not possess the *SCCmec* cassette while K07-204 possessed *SCCmecIVc(2B)*. Out of 29 human isolates of ST72 *S. aureus*, 15 isolates showed the presence of *SCCmec* cassette ranging from type *IV(2B)*, *IVa(2B)*, *IVc(2B)* to *V(5C2)*. The presence of *mecA* gene was identified in two ST72 isolates (E16SA093 and F17SA003), but no typable *SCCmec* cassette was identified (Orange box in Figure 1B). The presence of arginine catabolite mobile element (ACME) adjacent to the *SCCmec* plays a significant role in the pathogenesis of SAUSA300 (Diep et al., 2008). The variation in ACME was classified into three types based on the presence and absence of arginine deiminase (*arc*) and oligopeptide permease (*opp3*; Miragaia et al., 2009). The ACME-I contains both the *arc* and *opp3*; while the ACME-II and ACME-III contain *arc* and *opp3*, respectively (Diep et al., 2008; Miragaia et al., 2009). The ACME was found to be absent in K07-561 while K07-204 comprises the ACME-II compared to the presence of ACME-I in both ST8-MRSA (USA300), and ST72 CV20 isolates (Diep et al., 2008; Miragaia et al., 2009; Shore et al., 2011).

Aminoglycoside modifying enzymes (AMEs) catalyze modification of different −OH or −NH_2_ groups of the 2-deoxystreptamine nucleus or sugar moieties and can be attained by nucleotidyltranferases, phosphotransferases, or acetyltransferases. Some isolates of ST72 possess the *aadD* gene, encoding a 4′,4″ adenyltransferase AME responsible for resistance against kanamycin/neomycin, paromomycin, and tobramycin (Figure 1A). The *aadD* resistance determinant was found to be located on a multicopy plasmid, pUB110, and was also detected in the chromosome of some *S. aureus* due to mutation of its origin of replication and integration through IS257 (Byrne et al., 1991). Aminoglycoside O-phosphotransferases (APHs) catalyze the transfer of a phosphate group to an aminoglycoside. Only the APH(3′′) subclass of enzymes mediates resistance to streptomycin (Ramirez and Tolmasky, 2010). The *aph(3')-III*, which encodes an enzyme responsible for phosphotransferase modification and consequent streptomycin resistance, was found in three isolates of ST72 (Figure 1A). ST72 isolates were also analyzed for the presence of a bifunctional aminoglycoside-modifying enzyme with acetylation and phosphotransferase activities encoded by the gene *aac(6')/aph(2')* (Figure 1A), which is known to provide a higher level of resistance against all aminoglycoside antibiotics except streptomycin. The *aac(6')/aph(2')* gene is most likely evolved by fusion of two genes and is located on a Tn*4001*-like transposon widely distributed among enterococci and staphylococci.

Macrolide resistance is typically due to the acquisition of genes coding for rRNA methylases, macrolide efflux pumps, and/or enzymatic inactivation (Roberts et al., 1999; Sutcliffe and Leclercq, 2002). Resistance to erythromycin is characteristically linked to resistance to other macrolides. The *ermC* gene encodes a methyltransferase that alters the ribosomal target site. This gene was present in ~17% of ST72 clones isolated from various locations (Figure 1A). Additionally, the *msr(A)* gene, which encodes an ATP-dependent efflux pump (ABC transporter) that confers resistance to macrolide-lincosamide-streptogramin (MLS) antibiotics, was also present in various ST72 isolates (Figure 1A). The *mph(C)* gene is known to confer erythromycin resistance by phosphorylation and is also responsible for spiramycin resistance (Matsuoka et al., 2003; Chesneau et al., 2007). Both the *msr(A)* and *mph(C)* genes are present sporadically in three ST72 isolates (Figure 1A). The *tetK* gene encoding the tetracycline efflux pump is occasionally present in ST72 isolates and can be incorporated in other isolates through Tn*10* acquisition (Truong-Bolduc et al., 2015). HA isolates can also develop resistance by target site protection. Genomic analysis of various ST72 isolates also revealed the presence of *dfrG*, which is a classic example of target replacement and is responsible for trimethoprim resistance (trimethoprim-resistant dihydrofolate reductase, DHFR). Genes responsible for fusidic acid resistance are *fusC* or *fusB* (~45% identity) that encodes a ribosome clearance protein (Cox et al., 2012) or activate major facilitator superfamily (MFS) efflux pump. In general, clinical bacterial isolates possess two types of antimicrobial resistance: (1) intrinsic resistance and (2) acquired resistance (Blair et al., 2015; Munita and Arias, 2016; Peterson and Kaur, 2018). Our findings and the results from previous studies clearly indicate that ST72 isolates appear to be continually acquiring various antibiotic genes from various resistant or antibiotic-producing microbes in the environment by horizontal gene transfer and are enriched by clonal selection due to the antibiotic pressure exerted in the hospital setting.

#### Validation of Antimicrobial Resistance in K07-204 and K07-561 ST72 Isolates

**Figure 2:**
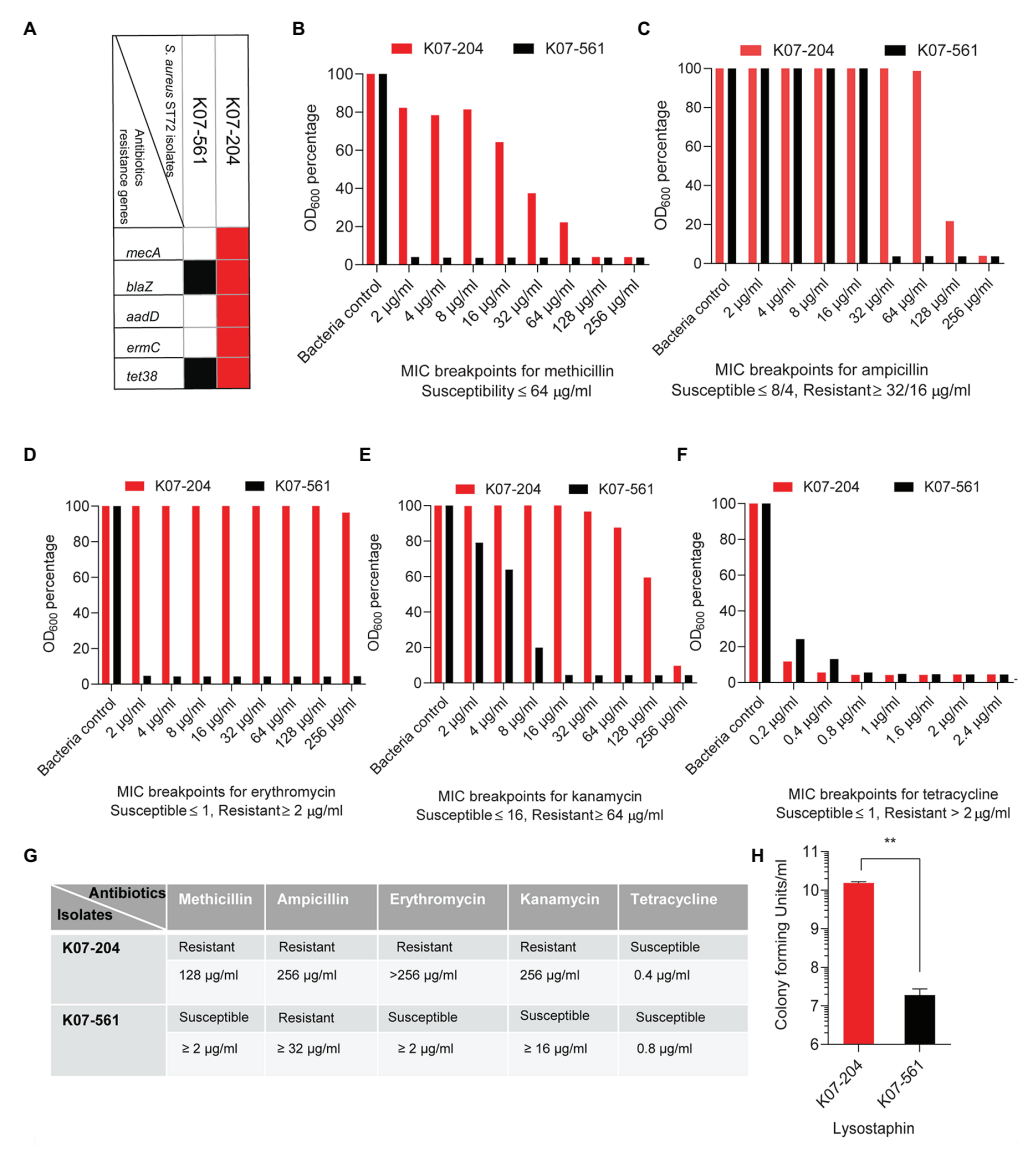
Experimental validation of the antimicrobial resistance/susceptibility of the human ST72 isolates K07-204 and K07-561. **(A)** The diagram shows isolate-specific antibiotic resistance gene where the presence of a gene is denoted by a red and black box; **(B)** Methicillin susceptibility of K07-561 (MSSA) and resistance of K07-204 (MRSA); **(C)** Variable levels of ampicillin resistance of the two isolates; **(D)** Erythromycin resistance of K07-204 and susceptibility of K07-561; **(E)** Variable levels of kanamycin resistance of K07-204 and susceptibility of K07-561; **(F)** Tetracycline susceptibility of two isolates according to European Committee on Antimicrobial Susceptibility Testing (EUCAST) clinical breakpoint table despite the presence of a major facilitator superfamily efflux pump (MFS) *tet38*; **(G)** Summary of the resistant/susceptible phenotypes of ST72 isolates (K07-204 and K07-561) and their corresponding MIC_90_; and **(H)** Lysostaphin resistance of K07-204 (*lys^r^*) in comparison to the lysostaphin sensitivity of K07-561(*lys^s^*) as reported earlier (Batool et al., 2020a).

Genome analysis of K07-204 indicated the presence of antibiotic resistance genes *mecA*, *blaZ*, *ermC*, and *aadD* while K07-561 possessed only the *blaZ* gene in its genome (Figure 2A). Additionally, whole genome sequence analysis of both human isolates showed the presence of *tet38* that encodes a major facilitator superfamily (MFS) efflux pump responsible for the efflux of tetracycline (Figure 2A). To verify the genotypic data, we tested susceptibility to methicillin, ampicillin, erythromycin, kanamycin, and tetracycline according to CLSI and EUCAST clinical breakpoints. ST72 K07-204 showed greater resistance to methicillin (MRSA) than K07-561 (Figure 2B). Furthermore, due to the absence of the *mecA* gene, K07-561 was extremely susceptible to methicillin (MSSA; Figure 2B). The widespread presence of *blaZ* (~86%), which encodes a β-lactamase that can hydrolyze β-lactam antibiotics, was tested by treating the two human isolates with varying concentrations of ampicillin. Both K07-204 and K07-561 showed resistance to ampicillin (Figure 2C). In addition to methicillin, K07-204 also showed resistance to erythromycin while K07-561 was susceptible to erythromycin (Figure 2D). ST72 K07-204 was found to be kanamycin resistant while K07-561 showed the susceptibility against kanamycin (Figure 2E). Consistent with the genome analysis showing the presence of a major facilitator superfamily (MFS) efflux pump *tet38*, both isolates were found to be susceptible to tetracycline according to EUCAST clinical breakpoint table (Figure 2F). The summary of the resistant/susceptible phenotypes of ST72 isolates (K07-204 and K07-561) and *S. aureus* USA300 (Supplementary Figure S1) and the corresponding MIC_90_ of tested antibiotics is shown in Figure 2G. Due to MDR, we tested the resistance of ST72 isolates to lysostaphin. Surprisingly, K07-561 showed lysostaphin susceptibility (*lys^s^*) while K07-204 was ~1000 times lysostaphin resistant (*lys^r^*) as compared to K07-561 (Figure 2H; Batool et al., 2020a). These two ST72 isolates were obtained from human subjects; their differential response to lysostaphin makes these two isolates an attractive model to elucidate mechanisms of enzybiotic resistance to lysostaphin (GenBank acc. no. JACSIU000000000.1 and JACORE000000000.1; Batool et al., 2020b). The ST72 isolates showed resistance to a wide range of antibiotics, most likely due to continuous acquisition of new antibiotics resistance genes or accumulation of mutations, and their biofilm formation ability suggests they can potentially be important contributors to nosocomial infections.

### Genotypic and Phenotypic Studies of Virulence Potential in Human Isolates of ST72

#### Comparative Genetic Distribution of ST72 Isolate-Specific Virulence Genes

First, we identified virulence factors commonly present in the 29 ST72 isolates including K07-204 and K07-561, and *S. aureus* USA300 (GenBank Accession no. CP012119) ST8 isolate (Supplementary Table S1). Then, we identified ST72 isolate-specific virulence factors by subtracting the common virulence genes present in all isolates from the total virulence genes (Figure 1A; Supplementary Table S1). The virulence factor *adsA* encodes adenosine synthase A, a cell wall-anchored enzyme that converts adenosine monophosphate to adenosine (Thammavongsa et al., 2009; Winstel et al., 2018). This gene was present in K07-204 along with the 27 other ST72 strains analyzed and ST8 (USA300 clone), but was absent in K07-561 (Figure 1A), indicating that K07-561 may use a different mechanism to evade the host immune system to establish infection. The gene *aur* encodes a metalloproteinase referred to as an aureolysin (Sabat et al., 2000) that cleaves complement C3 to mediate immune evasion (Laarman et al., 2011), thereby allowing *S. aureus* to establish an infection. The *aur* gene was present in K07-204 and the 27 other ST72 isolates but was absent in K07-561 (Figure 1A). The *clfA* encoding microbial surface components recognizing adhesive matrix molecules (MSCRAMM) clumping factor (ClfA) interacts with the γ-chain of fibrinogen. ClfA is present in most clinical isolates of *S. aureus* and is known to be responsible for the clotting of blood plasma, which has been implicated in septic arthritis and infective endocarditis (Ganesh et al., 2008). The *clfA* was present in ~58% of the ST72 isolates but absent from both K07-204 and K07-561 (Figure 1A). The *hysA* gene encodes a hyaluronidase that primarily degrades hyaluronic acid, a major component of the extracellular matrix (ECM) of human tissues. The protein encoded by *hysA* has previously been determined to be a potent virulence factor as a *hysA* knockout showed a 4-log_10_ value reduction in bacterial burden in a neutropenic, murine model of pulmonary infection (Ibberson et al., 2014). The *hysA* virulence factor was found to be present in all ST72 except for K07-561 (Figure 1A). Biofilm formation by staphylococcal cells is closely related to their pathogenic potential especially in endocarditis and osteomyelitis and contributes to their ability to grow on implants and medical devices. The main constituent of biofilms is polysaccharides such as N-acetyl glucosamine, which is the product of the *icaABCD* operon, which itself is negatively regulated by *icaR* (Cramton et al., 1999; Cue et al., 2012). The *lip* gene encodes glycerol ester hydrolase, which is a fatty acid modifying enzyme that inactivates bactericidal lipids secreted by the host at the site of abscesses, and therefore enhances the growth of staphylococcal cells (Shryock et al., 1992; Lowe et al., 1998). The *icaABCDR* operon and *lip* were present in all ST72 isolates except K07-561 (Figure 1A). Leucocidin targets immune cells and creates pores, causing cell death (Pédelacq et al., 1999; Vandenesch et al., 2012). In general, CA-MRSA ST72 isolates are considered to be Panton Valentine leucocidin (PVL) negative (Joo et al., 2016). However, of the 29 ST72 isolates analyzed in the current study, three isolates of ST72 possessed both, *lukF-PV*, and *lukS-PV* (Figures 1A,B). Adhesion of the bacterium to the host ECM is a prerequisite for successful infection. Cell wall-anchored staphylococcal adhesins form a family of MSCRAMM encoded by *sdr* genes (Sabat et al., 2006). Genes encoding serine-aspartate repeat Sdr proteins (*sdrC* and *sdrE*) were present in most (>70%) ST72 isolates (Figure 1A), suggesting that these isolates can bind efficiently to the host ECM. We next assessed the distribution of staphylococcal protein A (*spA*) that binds immunoglobulin G (IgG) to overcome the host defense system (Brignoli et al., 2019). The *spA* gene was sporadically (~27%) present in ST72 isolates including K07-204 and K07-561 (Figure 1A). Staphylococcal enterotoxin-like (sel) proteins (Argudín et al., 2012) are variably present in ST72. However, Sel proteins were found to be absent in both K07-204 and K07-561 isolates (Figure 1A). The toxic shock syndrome toxin gene (*tsst-*1) encodes a protein that belongs to the superantigenic toxin family that stimulates T-cells and have a particular Vβ element in their T-cell receptors (Omoe et al., 2005). TSST-1 is an extracellularly secreted staphylococcal enterotoxin (SE) that causes fever, rash, and multiorgan failure. Of the 29 isolates of ST72, TSST-1 was absent from 27 isolates including K07-204 and K07-561 (Figure 1A). Coagulases acts as determinants of a protective immune response against *S. aureus* (McAdow et al., 2012). Staphylococcal cells secrete two coagulases, namely Coa and von Willebrand factor binding protein (vWbp). These coagulases activate host prothrombin, which converts fibrinogen to fibrin and induces blood clotting. Coagulases play an important role in the pathophysiology of *S. aureus* during infective endocarditis, sepsis, and pneumonia. vWbp was absent from all studied 29 ST72 isolates (Figure 1A). It is noteworthy that the “presence/absence” and “percentage of occurrence” of an antibiotic or virulence gene in a sequence type may vary depending upon the “time of isolation,” and “number of isolates taken for the analysis,” respectively.

#### *In vitro* and *in vivo* Validation of Genomic Findings of the Virulence Potential for the Two ST72 Isolates

**Figure 3:**
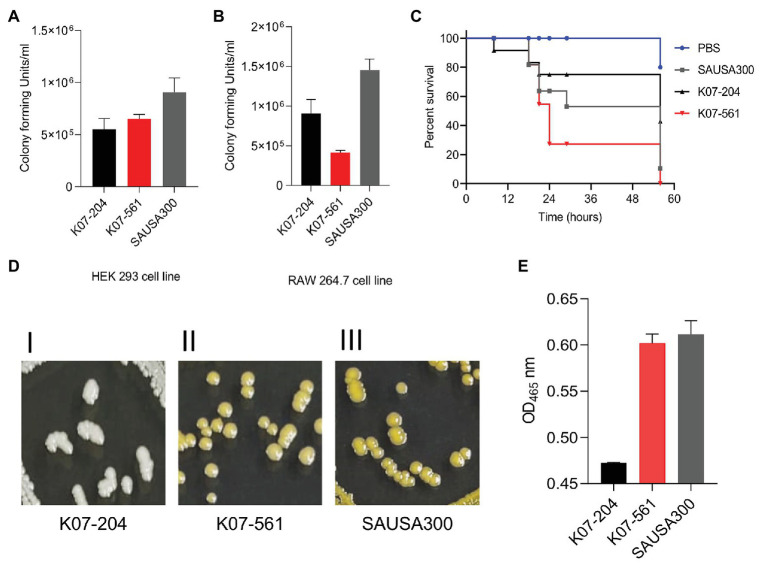
Comparative study of the infection potential of K07-204 and K07-561 relative to that of ST8 (*S. aureus* USA300) under *in vitro* and *in vivo* infection conditions, and the possible involvement of major virulence factors. **(A,B)** K07-204, K07-561, and *S. aureus* USA300 showing the differential invasion potential in HEK293 cells **(A)**, and phagocytosis and intracellular survival in murine macrophage 264.7 cells **(B)**. **(C)** *In vivo* wax-worm infection model showing the comparative pathogenesis of K07-204 and K07-561 with *S. aureus* USA300. The pathogenesis potential of K07-561 was higher than that of *S. aureus* USA300. **(D,E)** Qualitative and quantitative assessment of staphyloxanthin in K07-204 and K07-561 and comparison with *S. aureus* USA300; K07-204 formed white colonies whereas K07-561 and *S. aureus* USA300 formed yellow colonies on a TSB agar plate **(D)**; and **(E)** Quantitation of extracted staphyloxanthin at A_465 nm_. Both K07-561 and *S. aureus* USA300 had about 20-fold higher staphyloxanthin levels than K07-204, which could be a possible reason for the equivalent pathogenesis potential of K07-561 and *S. aureus* USA300 under *in vivo* conditions.

To validate the subtractive genomics data that showed the presence of isolate-specific virulence factors (Figure 1), we compared the infection potential of human isolates K07-204 and K07-561 under *in vitro* mammalian cell culture conditions. The invasion potential of K07-204 and K07-561 was compared with that of SAUSA300 (ST8) in human embryonic kidney cells (HEK 293). The invasion potential of SAUSA300 was found to be slightly higher in comparison to the two ST72 isolates (Figure 3A). Out of 17 isolate-specific virulence factors analyzed, SAUSA300 possesses 15 virulence factors, while K07-204 and K07-561 comprised of nine and one virulence factors, respectively. In comparison to SAUSA300, six virulence factors (*clfA*, *lukF-PV*, *lukS-PV*, *selK*, *selq*, and *vWbp*) were found to be absent in both K07-204 and K07-561 ST72 isolates (Figure 1A). The absence of *clfA*, *lukF-PV*, *lukS-PV*, *selK*, *selq*, and *vWbp* could be plausible reason of reduced invasion potential of ST72 in comparison to SAUSA300. Furthermore, phagocytosis followed by intracellular survival of K07-204 and K07-561 in comparison to SAUSA300 was evaluated in murine macrophage cells (RAW 264.7). Intracellular survival of K07-561 isolate was found to be lower than that of K07-204 (Figure 3B). The AdsA protein plays a key role in immune system evasion by synthesizing cytotoxic deoxyadenosine (dAdo) from neutrophil extracellular traps to eliminate macrophages (Winstel et al., 2018). Interestingly, the AdsA protein was found to be absent in K07-561 (Figure 3B), could be one of the reasons of its lower survival in murine macrophage.

Isolated *in vitro* mammalian cell culture infection experiments usually do not reflect the challenges pathogens face when confronted with multiple host-induced stresses, wherein these challenges have to be overcome by pathogens to establish a successful infection. Therefore, we investigated the pathogenic potential of these two isolates using a wax-worm infection model, which is well-known to possess a strong immune system (Tsai et al., 2016; Cutuli et al., 2019). Remarkably, K07-561 possessed higher pathogenic potential than K07-204 under *in vivo* conditions (Figure 3C). To determine why the pathogenic potential of K07-561 was higher than that of K07-204, we assayed staphylococcal defense-virulence factors that protect staphylococcal cells from the host immune response during pathogenesis. Interestingly, we observed the differences in staphyloxanthin among isolates when observing their growth (Figure 3C). Staphyloxanthin is one of the important factors that protects staphylococcal cells from oxidative stress under *in vivo* infection conditions (Clauditz et al., 2006; Chen et al., 2016). The staphyloxanthin level of K07-561 was 20-fold higher than that of K07-204, which could be a contributing factor of higher survival potential of K07-561 during *in vivo* infection (Figures 3D,E). The lower level of staphyloxanthin is known to reduce the protection of staphylococcal cells under *in vivo* conditions against host’s consolidated immune response (Liu et al., 2008) which is likely to translate into a lower pathogenic potential of K07-204. It is noteworthy that most of the drug-developments are currently targeting the offensive-virulence factors (toxins)responsible for the pathogenesis, however, the current study showed that the defensive/protective virulence factor(s) [Staphyloxanthin, other reactive oxygen species (ROS) scavenging enzymes etc.] which provides the survival advantages to the pathogen under host or antibiotics stresses, create significant variability in virulence potential and disease outcomes.

### Phylogenetic Analysis of ST72 Isolates

**Figure 4:**
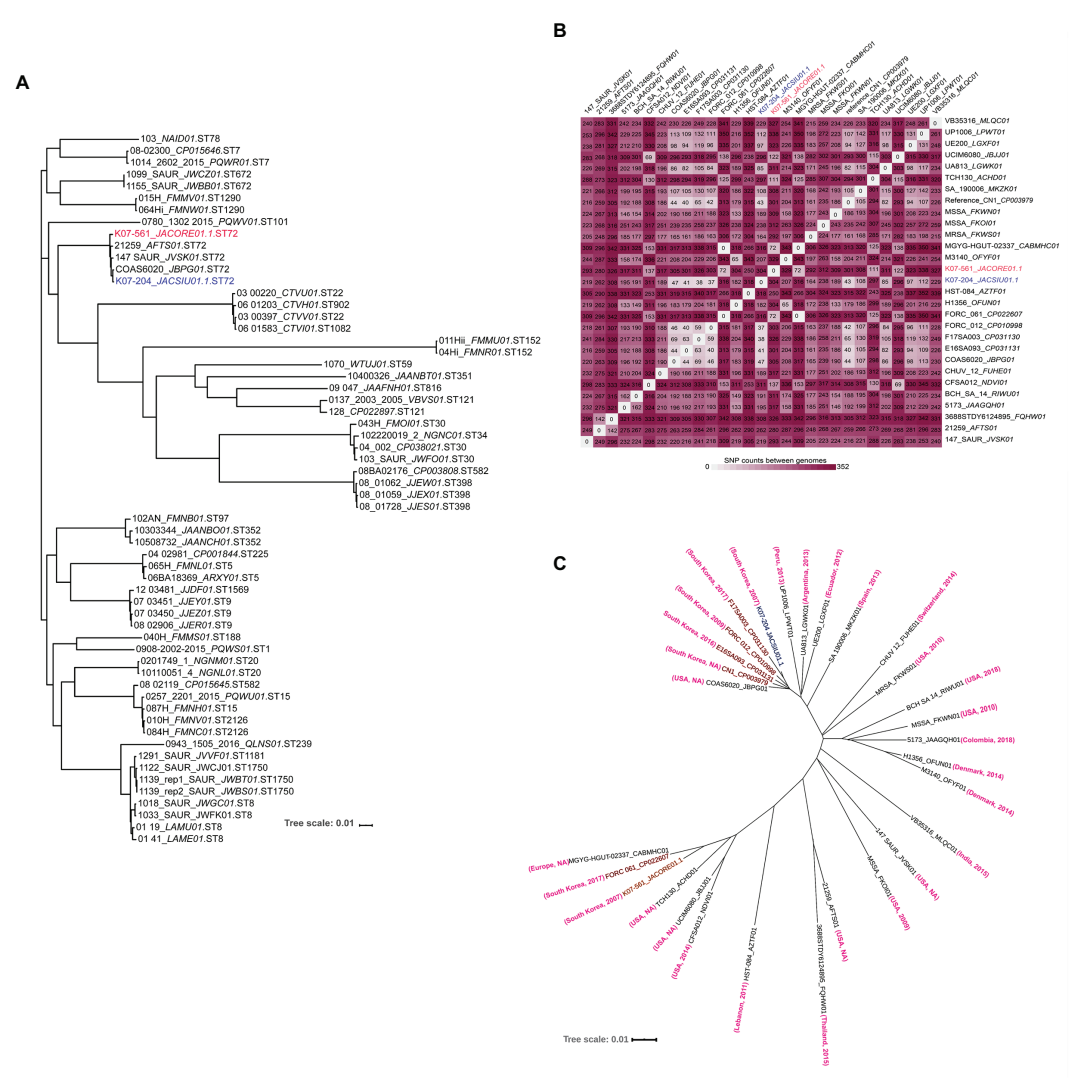
Phylogenetic analysis of various sequence types, a single nucleotide polymorphism matrix of ST72 isolates, and a phylogram of ST72 showing the phylogenetic affinities of K07-204 and K07-561. **(A)** Maximum-likelihood phylogeny of 60 isolates based on single nucleotide polymorphisms (SNPs) showing closely related sequence types of ST72. As expected, five representative ST72 isolates comprising K07-204 and K07-561 are grouped together among various sequence types. **(B)** The SNP matrix of 29 ST72 isolates showing variation among various ST72 isolates; K07-204 and K07-561 differed by 304 SNPs. **(C)** SNP-based phylogram showing the phylogenetic relationships of K07-204 (blue) and K07-561 (red) and their closely related 27 ST72 isolates wherein K07-204 is closely related with F17SA003 while K07-561 is closely related with two FORC_061 (Korea) and MGYG-HGUT-02337 (Isolation location: NA, submitter EMBL_EBI, Europe, 2019) as both these isolates has no variation with 0 SNP. The “country” and the “year of isolation” of ST72 isolates are shown in bracket wherein NA stands for “not available.”

Single nucleotide polymorphisms (SNPs) based phylogenomic analysis of 60 isolates of various sequence types of *S. aureus* including the five representative ST72 isolates were analyzed to determine the relatedness of ST72 with other sequence types as described earlier (Challagundla et al., 2018). The SNP phylogenetic tree of 60 isolates showed that ST72 isolates grouped in the same cluster irrespective of their location and time of isolation [COAS6070_JBPG01 (USA, 2013), 147_SAUR_JVSK01 (USA, 2014), K07-204_JACSIU000000000.1 (Korea, 2007), K07-561_JACORE000000000.1 (Korea, 2007) and 21259_AFTS01 (J. Craig Venter Institute, USA, 2011); (Figure 4A)]. This phylogenetic analysis also revealed that ST72 is closely related to ST22, ST902, ST101, and ST1082 (Figure 4A). The SNP-based phylogeny of ST72 with other isolates showed that the representative ST72 isolates are clustered together, and thus this analysis provided the closest relatives of ST72 (Figure 4A). Furthermore, the variability among the ST72 isolates was figured out by analyzing 29 ST72 genomes isolated from different locations and time by creating a matrix of SNPs using the CSI phylogeny wherein CN1 isolates of ST72 (GenBank Accession no. CP003979) was considered as reference genome (Figure 4B). Detailed analysis of the SNP matrix showed that the accumulation of SNPs in ST72 was highly variable across time and space ranging from zero between FORC_061_CP022607 (Korea, 2017) and MGYG-HGUT-02337_CABMHC01 (location: not disclosed, submitter EMBL_EBI, Europe, 2019) to 345 between HST_084_ AZTF01 (Lebanon, 2011) and M3140_OFYF01 (Denmark, 2014). Interestingly, both K07-204 and K07-561 were collected in 2007 from South Korea differed by 304 SNPs suggesting the existence of two distinct lineage at the point of isolation (South Korea, 2007) with different sets of antibiotic and virulence genes (Figure 4B). The accumulation of significant number of SNPs at the same time and place revealed that various ST72 isolates steered rapid acclimatization against challenges to adapt from CA-MRSA to one of the major MRSA in hospital settings. Furthermore, the SNP-based phylogram was created to analyze the relatedness of K07-204 and K07-561 among ST72 clones isolated across the globe (Figure 4C). On the other hand, the K07-204 is closely related to F17SA003_CP031130 with 38 SNPs although these isolates were collected from South Korea in 2007 and 2018, respectively. In the same way K07-561 is closely related to FORC_061_CP022607 with 72 SNPs even though these isolates were reported from South Korea in 2007 and 2017, respectively (Figure 4C). These results indicate that the SNPs as well as antibiotics and virulence genes are acquired differentially by CA-MRSA depending upon the challenges they faced with (hosts and antibiotic pressure, abiotic and biotic stresses) at various locations and adaptations to human hosts. The isolate-specific antibiotic resistance and virulence genes of various ST72 clones identified in this study can aid in assessment of current and future therapeutic options and rapid molecular diagnosis.

## Conclusions

Community-associated (CA) methicillin-resistant *Staphylococcus aureus* (MRSA) ST72 clones, which have been reported to have relatively low virulence, are predominant in Korea (Park et al., 2015). Recently, however, community-associated ST72 MRSA isolates have emerged as major hospital-associated isolates in Korea, accounting for 32% of total MRSA infections and causing mild to severe disease ranging from superficial skin wounds to lethal soft-tissue infections, pneumonia, bacteremia, sepsis, and endocarditis (Joo et al., 2016). CA-ST72 isolates generally do not produce the PVL toxin (Joo et al., 2016). However, the current study showed that some ST72 human isolates are PVL positive (Figure 1). The infection potential of K07-561 was found to be higher than that of *S. aureus* USA300 in an *in vivo* wax-worm infection model. Moreover, the K07-204 isolate was found to be resistant to multiple different classes of antibiotics, including one of the most potent staphylolytic enzymes, lysostaphin. From this study, we were able to identify isolate-specific antibiotic resistance and virulence genes that will help to determine the dynamics of gene acquisition, accumulation of SNPs, pave the way for future diagnostic studies and alternative therapeutic options for the treatment of highly pathogenic isolates of MDR ST72 MRSA.

## Data Availability Statement

The datasets presented in this study can be found in online repositories. The names of the repository/repositories and accession number(s) can be found in the article/Supplementary Material.

## Author Contributions

AC and KK have designed the study. NB, AS, and AC analyzed the data. The final draft is approved by all the authors.

### Conflict of Interest

The authors declare that the research was conducted in the absence of any commercial or financial relationships that could be construed as a potential conflict of interest.

## Footnotes

^1^https://cge.cbs.dtu.dk/services/CSIPhylogeny
