# Supplementary material for: Genome-Wide Analysis of Staphylococcus aureus Sequence Type 72 Isolates Provides Insights Into Resistance Against Antimicrobial Agents and Virulence Potential
Source: Front Microbiol. 2021 Jan 20;11:613800. doi: 10.3389/fmicb.2020.613800 (PMC7854921; doi:10.3389/fmicb.2020.613800)
Supplement: Supplementary file 1 [file Data_Sheet_1.pdf]

TITLE

Genome-wide analysis of *Staphylococcus aureus* sequence type 72 isolates provides insights into resistance against antimicrobial agents and virulence potential

RUNNING TITLE

Genomics: ST72 Resistance and Virulence

AUTHORS

Nayab Batool[1], Amen Shamim[1], Akhilesh Kumar Chaurasia[1, 2]*, and Kyeong Kyu Kim[1, 2, 3]*

[1]Department of Precision Medicine, Sungkyunkwan University School of Medicine, Suwon 16419, Korea

[2]Institute of Antimicrobial Resistance and Therapeutics (IAMRT), Sungkyunkwan University (SKKU), Suwon 16419, Korea

[3]Samsung Advanced Institute for Health Sciences and Technology (SAIHST), Samsung Medical Center (SMC), Sungkyunkwan University School of Medicine, Seoul 0635, Korea

CORRESPONDING AUTHORS

Akhilesh Kumar Chaurasia (chaurasia@skku.edu)

Kyeong Kyu Kim (kyeongkyu@skku.edu)

PHONE: 82-31-299-6152

FAX: 82-31-299-61

Table S1. Isolate-specific and common virulence factors of 29 ST72 and one ST8 isolates of *Staphylococcus aureus*⊕

SN*	Isolates	GenBank accession number^	Country	Year of Isolation/Genome Data	Specific virulence factors#	Common virulence factors$
1	K07-204	*JACSIU000000000.1*	South Korea	2007/2020	*adsA, aur, cap8L, chp, esaG1, esaG2,_esaG3*[2], *esaG6, esaG7, esaG8, hysA, lip, lukD, sak, sbi, scn, sdrC, sdrE, spa*	*cap8A, cap8B, cap8C, cap8D, cap8E, cap8F, cap8G, cap8M, cap8N, cap8O, cap8P, ebp, esaA, esaB, esaD, esaE, esaG4, essA, essB, essC, esxA, esxB, esxC, esxD, geh, hlb, hld, hlgA, hlgB, hlgC, hly/hla, icaA, icaB, icaC, icaD, icaR, isdA, isdB, isdC, isdD, isdE, isdF, isdG, srtB, sspA, sspB, sspC*
2	K07-561	*JACORE000000000.1*	South Korea	2007/2020	*cap8L, chp, esaG1, esaG2, esaG3, esaG6, esaG7, esaG8*[2], *sak, sbi, scn, spa*	
3	147_SAUR	*JVSK01*	USA: WA	NA/2020	*adsA, aur, cap8L, clfA, esaG3, esaG6, esaG7, hysA, lip, lukD, sak, sbi, scn, sdrC, sdrE*	
4	21259	*AFTS01*	USA	NA•/2011	*adsA, aur, cap8L, esaG1, esaG2, esaG3, esaG6, esaG8, hysA, lip, lukD, sak, sbi, scn, sdrC, sdrE, sec, sell, tsst-1*	
5	CN1	*CP003979*	South Korea	NA/2013	*adsA, aur, cap8L, chp, clfA, esaG1, esaG2, esaG3, esaG6, esaG7, esaG8, hysA, lip, lukD, sak, sbi, scn, sdrC, sdrE*	
6	COAS6020	*JBPG01*	USA: Irvine, CA	NA/2013	*adsA, aur, cap8L, chp, esaG1, esaG2,_esaG3, esaG6, esaG7, esaG8, hysA, lip, lukD, sak, sbi, scn, sdrC, spa*	
7	MGYG_HGUT_02337	*CABMHC01*	Isolation NA, Submitted from EMBL-EBI	NA/2019	*adsA, aur, cap8L, chp, esaG1, esaG2,_esaG3, esaG6, esaG7, esaG8, hysA, lip, lukD, sak, sbi, scn, sdrC, spa*	
8	TCH130	*ACHD01*	Isolation NA, Submitted from USA	NA/2013	*adsA, aur, cap8L, chp, esaG1, esaG2,_esaG3, esaG6, esaG7, esaG8, hysA, lip, lukD, sak, sbi, scn*	
9	UCIM6080	*JBJJ01*	USA: Irvine, CA	NA/2013	*adsA, aur, cap8L, chp, esaG1, esaG2,_esaG3, esaG6, esaG7, esaG8, hysA, lip, lukD, sak, sbi, scn, sdrC, sdrE*	

10	MSSA	*FKOI01*	USA	2009/2016	*adsA*, *aur*, *cap8L*, *clfA*, *esaG1*, *esaG2*,_*esaG3*, *esaG6*, *esaG7*, *esaG8*, *hysA*, *lip*, *lukD*, *sak*, *sbi*, *scn*, *sdrC*, *sdrE*	
11	FORC_012	*CP010998*	South Korea	2009/2016	*adsA*, *aur*, *cap8L*, *chp*, *clfA*, *esaG1*, *esaG2*, *esaG3*, *esaG6*, *esaG7*, *esaG8*, *hysA*, *lip*, *lukD*, *sak*, *sbi*, *scn*, *sdrC*, *sdrE*, *spa*	
12	MRSA	*FKWS01*	USA	2010/2016	*adsA*, *aur*, *cap8L*, *chp*, *clfA*, *esaG1*, *esaG2*, *esaG3*, *esaG6*, *esaG7*, *esaG8*, *hysA*, *lip*, *lukD*, *sak*, *sbi*, *scn*, *sdrC*, *sdrE*	
13	MSSA	*FKWN01*	USA	2010/2016	*adsA*, *aur*, *cap8L*, *chp*, *clfA*, *esaG1*, *esaG2*, *esaG3*, *esaG6*, *esaG8*, *hysA*, *lip*, *lukD*, *sak*, *sbi*, *scn*, *sdrC*, *sdrE*, *sec*, *sell*	
14	HST_084	*AZTF01*	Lebanon: Byblos	2011/2014	*adsA*, *aur*, *cap8L*, *clfA*, *esaG1*, *esaG2*,_*esaG3*, *esaG6*, *esaG7*, *esaG8*, *hysA*, *lip*, *lukD*, *sbi*, *sdrC*, *sec*, *sell*	
15	UE200	*LGXF01*	Ecuador: Quito	2012/2017	*adsA*, *aur*, *cap8L*, *chp*, *esaG1*, *esaG2*,_*esaG3*, *esaG6*, *esaG7*, *esaG8*, *hysA*, *lip*, *lukD*, *sak*, *sbi*, *scn*, *sdrE*	
16	SA_190006	*MKZK01*	Spain: Madrid	2013/2016	*adsA*, *aur*, *cap8L*, *chp*, *clfA*, *esaG1*, *esaG2*, *esaG3*, *esaG6*, *esaG7*, *esaG8*, *hysA*, *lip*, *lukD*, *sak*, *sbi*, *scn*, *sdrC*, *sdrE*	
17	UA813	*LGWK01*	Argentina: Buenos Aires	2013/2017	*adsA*, *aur*, *cap8L*, *chp*, esaG1, *esaG3*,_*esaG7*, *esaG8*, *hysA*, *lip*, *lukD*, *sak*, *sbi*, *scn*, *sdrE*	
18	UP1006	*LPWT01*	Peru: Lima	2013/2020	*adsA*, *aur*, *esaG1*, *esaG2*, *esaG3*, *esaG6*, *esaG7*, *esaG8*, *hysA*, *lip*, *lukD*, *sbi*, *sdrE*	
19	H1356	*OFUN01*	Denmark	2014/2018	*adsA*, *aur*, *cap8L*, *chp*, *clfA*, *esaG1*, *esaG2*, *esaG3*, *esaG6*, *esaG7*, *esaG8*, *hysA*, *lip*, *lukD*, *lukS-PV*, *lukF-PV*, *sak*, *sbi*, *scn*, *sdrC*, *sdrE*, *sec*, *sell*	

20	M3140	OFYF01	Denmark	2014/2018	*adsA*, *aur*, *cap8L*, *chp*, *clfA*, *esaG1*, *esaG2*, *esaG3*, *esaG6*, *esaG7*, *esaG8*, *hysA*, *lip*, *lukD*, *lukS-PV*, *lukF-PV*, *sak*, *sbi*, *scn*, *sdrC*, *sdrE*, *sec*, *sell*	
21	CFSA012	NDVI01	USA: Cincinnati	2014/2020	*adsA*, *aur*, *cap8L*, *chp*, *clfA*, *esaG1*, *esaG2*, *esaG3*, *esaG6*, *esaG7*, *esaG8*, *hysA*, *lip*, *lukD*, *sak*, *scn*, *sdrC*, *sdrE*, *spa*	
22	CHUV_12	FUHE01	Isolation NA, Submitted from Lausanne Switzerland	2014/2017	*adsA*, *aur*, *cap8L*, *chp*, *esaG1*, *esaG2*,_*esaG3*, *esaG6*, *esaG7*, *esaG8*, *hysA*, *lip*, *lukD*, *lukS-PV*, *lukF-PV*, *sak*, *sbi*, *scn*, *sdrC*, *sdrE*	
23	3688STDY6124895	FQHW01	Thailand	2015/2016	*adsA*, *aur*, *cap8L*, *clfA*, *esaG1*, *esaG6*, *hysA*, *lip*, *lukD*, *sak*, *sbi*, *scn*, *sdrC*, *sdrE*, *sec*, *sell*, *tsst-1*	
24	VB35316	MLQC01	India: Vellore	2015/2020	*adsA*, *aur*, *cap8L*, *esaG1*, *esaG2*, *esaG3*, *esaG6*, *esaG8*, *hysA*, *lip*, *lukD*, *sak*, *sbi*, *scn*	
25	E16SA093	CP031131	South Korea	20162018	*adsA*, *aur*, *cap8L*, *chp*, *clfA*, *esaG1*, *esaG2*, *esaG3*, *esaG6*, *esaG7*, *esaG8*, *hysA*, *lip*, *lukD*, *sak*, *sbi*, *scn*, *sdrC*, *sdrE*	
26	F17SA003	CP031130	South Korea	2017/2018	*adsA*, *aur*, *cap8L*, *chp*, *clfA*, *esaG1*, *esaG2*, *esaG3*, *esaG6*, *esaG7*, *esaG8*, *hysA*, *lip*, *lukD*, *sak*, *sbi*, *scn*, *sdrC*, *sdrE*	
27	FORC_061	CP022607	South Korea	2017/2018	*adsA*, *aur*, *cap8L*, *chp*, *clfA*, *esaG1*, *esaG2*, *esaG3*, *esaG6*, *esaG7*, *esaG8*, *hysA*, *lip*, *lukD*, *sak*, *sbi*, *scn*, *sdrC*, *sdrE*, *spa*	
28	5173	JAAGQH01	Colombia: Pereira	2018/2020	*adsA*, *aur*, *cap8L*, *chp*, *esaG1*, *esaG2*,_*esaG3*, *esaG6*, *esaG7*, *esaG8*, *hysA*, *lip*, *lukD*, *sak*, *sbi*, *scn*, *sdrC*, *sdrE*, *sec*, *sell*, *spa*	
29	BCH_SA_14	RIWU01	USA: Boston, MA	2018/2020	*adsA*, *aur*, *cap8L*, *chp*, *clfA*, *esaG1*, *esaG2*, *esaG3*, *esaG6*, *esaG7*, *esaG8*, *hysA*, *lip*, *lukD*, *sak*, *sbi*, *scn*, *sdrC*, *sec*, *sell*	

30	USA300_2014_C01	*CP012119*	USA: Georgia	2011/2019	*adsA*, *aur*, *cap8L*, *chp*, *clfA*, *esaG1*, *esaG2*, *esaG3*, *esaG7*, *esaG9*, *hysA*, *lip*, *lukD*, *lukS-PV*, *lukF-PV*, *map*, *sak*, *sbi*, *scn*, *sdrC*, *sdrD*, *sdrE*, *selk*, *selq*, *spa*, *vWbp*	

⊕The total virulence factors of these isolates were analyzed using BacWGSTdb server.

*Serial number 1-29: ST72 isolates; Serial number 30: ST8 isolate.

•NA: Not available

^All the GenBank accession numbers are hyperlinked.

$ Virulence factors commonly present in all 30 isolates.

Isolate-specific virulence factors *i.e.* Virulence factors specifically present only in designated isolates. Isolate-specific virulence factors were analyzed by using subtractive genomics.

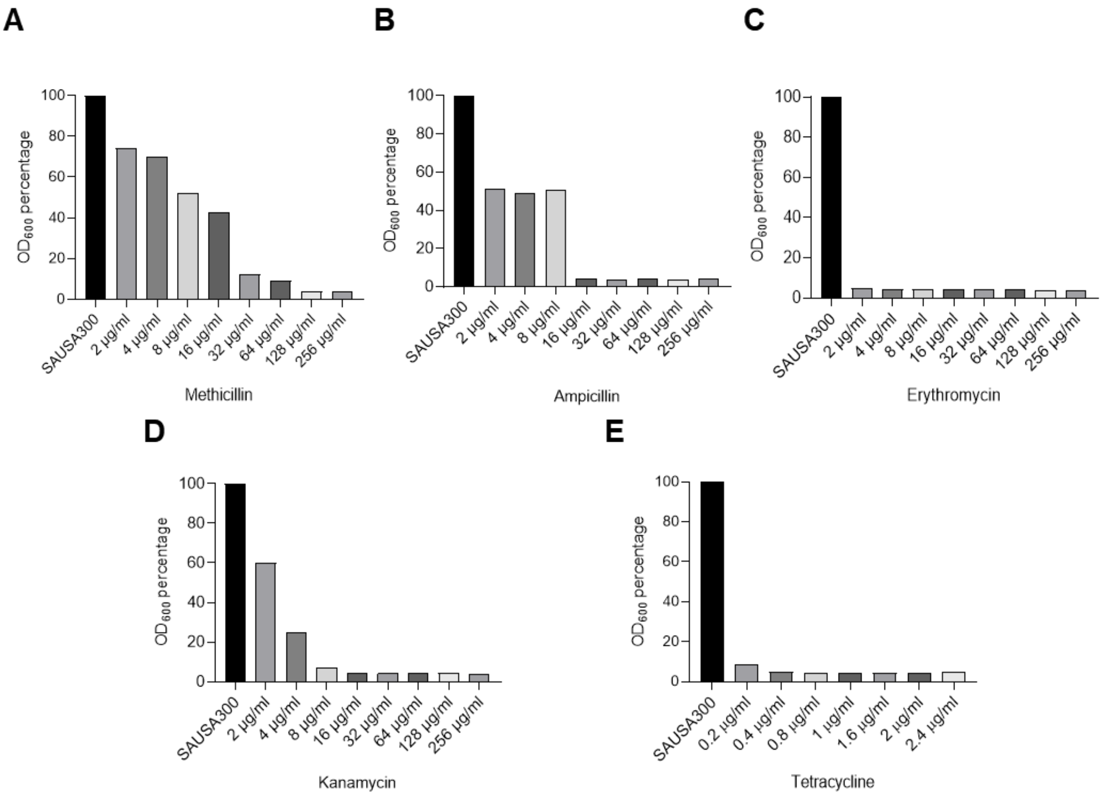

Figure S1. Antimicrobial resistance/susceptibility *S. aureus* USA300 FPR3757 against various antibiotics. (**A**) MIC_{90} of methicillin for SAUSA300 is ≤ 32 µg/ml; (**B**) MIC_{90} of ampicillin for SAUSA300 is ≤ 16 µg/ml; (**C**) MIC_{90} of erythromycin for SAUSA300 is ≤ 2 µg/ml; (**D**) MIC_{90} of kanamycin for SAUSA300 is ≤ 8 µg/ml; (**E**) MIC_{90} of tetracycline for SAUSA300 is ≤ 0.2 µg/ml.
